# The turnover of plant–frugivore interactions along plant range expansion: consequences for natural colonization processes

**DOI:** 10.1098/rspb.2022.2547

**Published:** 2023-05-31

**Authors:** Jorge Isla, Miguel Jácome-Flores, Juan M. Arroyo, Pedro Jordano

**Affiliations:** ^1^ Estación Biológica de Doñana, CSIC, Av. Americo Vespucio 26, 41092 Sevilla, Spain; ^2^ CONACYT-Centro del Cambio Global y la Sustentabilidad, 86080 Villahermosa, Tabasco, México; ^3^ Dept. Biología Vegetal y Ecología, Facultad de Biología, Universidad de Sevilla, 41012 Sevilla, Spain

**Keywords:** mutualistic interactions, plant range shifts, seed dispersal, ecological networks, reproductive ecology, frugivory

## Abstract

Plant–animal mutualisms such as seed dispersal are key interactions for sustaining plant range shifts. It remains elusive whether the organization of interactions with seed dispersers is reconfigured along the expansion landscape template and, if so, whether its effects accelerate or slow colonization. Here we analyse plant–frugivore interactions in a scenario of rapid population expansion of a Mediterranean juniper. We combined network analyses with field surveys, sampling interactions between individual plants and frugivores by DNA-barcoding and phototrapping over two seasons. We assess the role of intrinsic and extrinsic intraspecific variability in shaping interactions and we estimate the individual plant contributions to the seed rain. The whole interaction network was highly structured, with a distinct set of modules including individual plants and frugivore species arranged concordantly along the expansion gradient. The modular configuration was partially shaped by individual neighbourhood context (density and fecundity) and phenotypic traits (cone size). Interaction reconfiguration resulted in a higher and more uneven propagule contribution, with most effective dispersers having a prominent role at the colonization front stand, where a distinct subset of early arriving plants dominated the seed rain. Our study offers new insights into the key role of mutualistic interactions in colonization scenarios by promoting fast plant expansion processes.

## 1. Introduction

Plant–animal interactions are the cornerstone of many ecosystem functions, crucially determining the persistence of biodiversity [1,2]. These interactions are relentlessly shaped and reconfigured by the ecological context in which they occur, leading to variations in their outcomes [3]. For example, interactions may vary in terms of likelihood of occurrence, frequency, sign and strength due to factors such as climate change [4,5], elevational range [6], landscape fragmentation [7] and during plant range shifts [8]. Of strong interest is the role of interactions in plant range shifts, given the rapid changes that natural systems are currently facing [9]. In such scenarios the ability of plant populations to provide propagules is essential to withstand and colonize new habitats [10]. Approximately 70–90% of known woody species depend on vertebrate animals for seed dispersal [11], highlighting the critical role of plant–frugivore interactions in colonization processes and plant range shifts [12].

Range shifts occur due to the combined and consistent responses of local populations to a given global change driver. However, the reconfiguration of mutualistic interactions at local scales during the expansion process remains unclear. Recent studies demonstrate that antagonistic plant–animal interactions can be reshaped during local natural expansion scenarios, and that focusing on this scale is important to bridge interaction shifts with their demographic effects [8]. Studying the reconfiguration of interactions with seed dispersers at the population level over small scales may be key to understanding how this process occurs, providing an empirical link between shifts in interaction network topologies, with the demographic and vegetation dynamics occurring during range shifts.

Ecological networks have proven to be an effective theoretical framework and analytical tool for the study of biodiversity architecture [13]. While ecological networks have traditionally focused on species–species interactions, other organizational hierarchies may be more appropriate for addressing some ecological questions [14]. In fact, individuals, not species, are the actors in natural interactions, and therefore, a sub-specific approach can avoid underestimating inter-individual variability, which can be more significant within a species than between species [15]. Additionally, the individual scale allows for an effective link between network structure and demographic effects. In seed dispersal studies with a phytocentric perspective, individual-based bipartite networks can consist of two different groups (modes) of nodes. A particular plant species is typically represented by individual plants, whereas animals can be represented either by individuals (individual–individual interactions) or species (individual–species interactions). Previous research has shown that this individual scale is a powerful approach for examining various issues in seed dispersal interactions, such as intraspecific specialization–generalization processes [16–18], fitness consequences [17,19] and network configurations [20]. Intraspecific variability in interactions between plants and seed dispersers can arise from various intrinsic and extrinsic drivers. Plant phenotypic traits are the source of intrinsic variation, with individual fecundity (crop size) and fruit traits being the main drivers of variation [21]. Regarding the extrinsic context, variation in neighbourhood composition is also a strong driver of intraspecific differences in seed dispersal [21].

An ecological context, such as a natural shift in plant range, represents a source of inter-individual variability resulting in variation in plant stand characteristics, individual density, spatial coverage or demographic and genetic structure along the gradient between mature stands and the colonization front [22,23]. This intraspecific variability can drive topological patterns of interactions that have the potential to reshape the interaction networks. For instance, modularity is a pervasive property of interaction networks that could reflect habitat heterogeneity [24]. In an individual-based seed dispersal network, modules correspond to groups of individual plants sharing a higher number of interactions with similar frugivore species when compared to other plant groups. Recent analyses of modularity in individual-based seed dispersal networks revealed clusters of closely linked plant–animal nodes consistent with the ecological context in which the plants and animals are found. For example, Vissoto *et al.* [16] documented distinct subsets of individual plants forming modules with frugivorous species, based on their use of different habitats by the animals. In a similar approach, Friedemann *et al.* [20] found that, along a gradient of rainforest habitats, the modular clustering between frugivores and individual palms was largely due to habitat context rather than individual plant traits. Whether the organization of mutualistic interactions during natural plant expansion follows consistent, generalizable patterns remains unknown [25]. Assessing mutualistic interaction turnover and its functional consequences, such as propagule contribution, along plant range shifts will help us understand how plant expansion processes occur in nature.

In the context of plant–seed disperser interactions, the main outcome from the plant's perspective is seed movement from the mother plant to other locations. To accurately estimate the number of seeds actually dispersed from an individual plant, a comprehensive understanding of the factors that affect this process is crucial. Details of the interaction such as characteristics of the species–species visit and the seed treatment during ingestion or digestion are essential to obtaining reliable estimates of seed dispersal [26]. Additionally, an individual-based approach that incorporates data on the interaction between individual plants and frugivores, as well as plant phenotypic traits such as seed viability or the number of seeds per fruit, is necessary for estimating the individuals' seed contributions [17]. In natural settings, individual variation in seed contribution to the pooled seed rain is primarily driven by differences in the composition of the frugivore assemblages among plants [27]. For example, plants that attract diverse frugivore assemblages may have an advantage [28], although a small and effective assemblage may also suffice for successful dispersal [29]. Reconfigurations of individual assemblages can have significant implications for seed dispersal and the contribution that individual plants can make to the process of plant-range expansion. In turn, the variability in these contributions can provide insight into how fruit resource selection by frugivores drives the processes underlying range expansion in plants. For example, in situations where individual contributions are very similar, as determined by high similarity in frugivore assemblages, a homogeneous expansion may occur, leading to a continuous spread by diffusion and resulting in a ‘wave front' [30]. Alternatively, when the action of frugivores is uneven, only a small and distinct subset of individual plants may contribute most of the seed rain. Such strongly unequal contributions of propagules during pollination and seed dispersal have been repeatedly documented for plant populations [19,31,32]. During a local expansion process, this unequal distribution of individual contributions may be evidence of long-distance dispersal events [33]. These strongly dispersing individuals may serve as pioneer plants that colonized new areas, and contribute disproportionately to local dispersal, and colonization [34,35]. By tracking individual seed contribution and disperser assemblages along expansion gradients, it is possible to infer how these expansion processes are occurring.

In this study we assess how an individual-based plant–animal interaction network is re-structured by intrinsic and extrinsic factors along a plant expansion process. We also explore the functional consequences of recorded interactions in terms of individual seed contributions. In addition to the above objectives, we believe that analysing patterns of interaction in these contexts can provide clues about the expansion history of natural populations. Our study system consists of a *Juniperus phoenicea* subsp. *turbinata* population and its assemblage of seed-dispersing birds and mammals in the Doñana National Park (Spain). Species of the genus *Juniperus* are considered as foundation species in Mediterranean ecosystems. The protection of this area five decades ago has facilitated the rapid expansion of this species, generating a natural gradient of plant range expansion from mature stands to colonization fronts in the surrounding shrublands [36]. Recent research in the same system indicates that this rapid expansion has not been facilitated by an antagonistic release [8], suggesting that seed dispersers may have reconfigured their interactions across these landscapes, promoting rapid expansion from the colonization fronts. This ecological context, combined with the diverse assemblage of birds and mammals known for similar juniper species [37–39], provides an optimal study system to address the following objectives: (i) to evaluate the individual-based frugivory network of *J. phoenicea* configuration along a plant range expansion gradient; (ii) to explore the role of intraspecific variability and neighbourhood context in the observed network structure; (iii) to estimate individual contributions to seed dispersal along the gradient and its drivers; and finally (iv) to explore how this current picture of individual-based interactions along the gradient may provide us with clues about the expansion history.

## 2. Material and methods

### (a) Study species and area

*Juniperus phoenicea* subsp. *turbinata* (Cupressaceae) is a large shrub inhabiting coastal dunes and rocky habitats in the western Mediterranean [40]. Adult trees have an extended fruiting period (September–May) and produce fleshy brown–red cones, also called galbules (electronic supplementary material, figure S1). *Juniperus phoenicea* cones average five seeds per cone (s.d. = 1.17, range 1–10) and low seed-viability rates (mean ±1 s.d.) of 32 ± 14% viable seeds. Cones act functionally as fleshy fruits and are a key resource for several wintering migrant species like song thrush (*Turdus philomelos*) [37] and generalist mammals such as red fox (*Vulpes vulpes*, electronic supplementary material, figure S2) and badgers (*Meles meles*) [41].

The study was carried out in the Doñana National Park in SW Spain (see electronic supplementary material, figure S3). We selected three 1 ha stands along the juniper natural regeneration gradient. All the individual junipers were identified and georeferenced. The mature stand, named ‘*Sabinar del Marqués'* (MAR), has the highest juniper density and is an old forest with a high dominance of *J. phoenicea* (882 individuals/ha). Advancing towards the colonization front, the second stand called ‘*Sabinar del Ojillo’* (OJI) represents a stage of intermediate maturity with a lower juniper density (700 individuals/ha) and composed of Mediterranean scrubland and scattered pine trees. The last stand is named *‘Sabinar de Colonización*' (COL) and represents a colonization front or expansion area, which is characterised by a low density of scattered individuals (126 individuals/ha). Besides variation in juniper density and intraspecific composition among stands, there are also deterministic differences in individual phenotypes along the maturity gradient. Plants increase in size (height and cover), fecundity (crop size) and cone size toward the colonization front (electronic supplementary material, figure S4). In order to select individual plants uniformly distributed within each stand, we established five evenly distributed subplots in each stand (mean ± s.d.) of 522 ± 115 m^2^ subplot area. We used these subplots to randomly select 35 focal individual plants in each stand (*N* = 105). This protocol allowed us a stratified-random selection of the focal plants, ensuring a homogeneous distribution of individuals throughout the three stands. Fieldwork was carried out during two consecutive fruiting seasons (October 2018–May 2019 and September 2019–May 2020).

### (b) Frugivore interactions survey

Interactions between focal plants and seed dispersers were sampled using two complementary methods. We combined camera-trap monitoring of animal visits with DNA-barcoding analysis of faecal samples collected beneath the plants. The combination of sampling techniques is especially useful for monitoring diverse assemblages where each method is more adequate for specific interactions [42].

Eight camera-traps were placed and rotated periodically through the 35 focal plants in each area. Camera-traps were active for 10 days in each focal plant, and then relocated to the other eight individuals, thus ensuring that the 105 plants were sampled every fifty days. Cameras were fitted at 1 m above ground and 2–3 m from the focal plant, focusing on both ground and the 2/3 lower canopy of the plant to detect visits on the plant and from the ground. The cameras were active day and night, in video mode, with 10 s per video and 2 s between recordings. All camera-trap records were inspected to determine if they contained frugivore visits. All those detections in which the animal was only walking/flying, or showed a behaviour different from a cone-foraging visit (e.g. passive perching or scent marking) were not recorded as visitation events, representing a 52% reduction in the records dataset. Each pairwise interaction (individual-species) was standardized by sampling effort (time and plant cover area; see Quintero *et al.* [42]). Because the cameras were unable to record the whole plant, we weighted the interactions by the percentage of the recorded plant surface. In the field, we estimated that the camera recorded 60% of the surface for small plants (less than 20 m^2^ cover), 40% for medium plants (20–40 m^2^ cover) and 20% for large plants (greater than 40 m^2^ cover). For time standardization, we used the percentage of days recorded per plant with respect to the entire study. The total sampling duration was 434 days, averaging (mean ±1 s.d.) 82 ± 27 recording days per individual. For example, for a small plant with four visits by *Turdus merula*, we estimate 6.7 visits to the whole plant area. Since this plant was recorded during 20% of the study duration, we estimated a total of 33.3 *T. merula* visits to that individual in the whole study duration.

We used DNA-barcoding to identify the bird and mammal species that visit focal plants by collecting scats (or regurgitated seeds) in seed traps beneath the 105 individual junipers. One seed trap was placed per plant, except for the largest plants where two trays were installed to maximize the area covered. Additionally, we delimited a rectangular soil surface next to the seed trap where we also collected scats to increase the sampled surface. The area sampled under each individual was (mean ± 1 s.d.) 0.56 ± 0.1 square metres. We followed the sample collection, DNA extraction and PCR protocols described in González-Varo *et al.* [43], (see ‘DNA-Barcoding’ section in the electronic supplementary material). Reamplification was required for 16% of the collected samples (*n* = 2993). We finally obtained species identification for 92.6% of the samples (*n* = 2772). As in camera-trap sampling, we standardized the number of detected visits based on area and time. We calculated the percentage of plant canopy covered by the sampled surface beneath the plant to scale the interactions to the whole plant. For time standardization, we used the number of days during which the tray was actively collecting faeces relative to the whole study duration. From the 434 days of sampling duration, the number of sampling days per individual averaged (mean ±1 s.d.) 415 ± 8. See electronic supplementary material, table S1 in for individual sampling effort details.

By standardizing both datasets to the same units of time and area, we were able to merge them as an adjacency matrix to get the final interaction matrix [42]. Each cell of this matrix represents the estimated number of visits of each species to each individual plant for the two reproductive episodes pooled. We used this matrix to construct the individual-based weighted network between the 105 individual junipers and the frugivores that visit them.

### (c) Plant characteristics

Individual phenotype and neighbourhood context were thoroughly sampled for the 105 focal juniper individuals. Following Sallabanks [44], we classified plant attributes hierarchically. Individual intraspecific neighbourhood context was described by the juniper density and cone productivity in a buffer of 100 m^2^. Moving down the hierarchy, from each individual that we measured in the field we recorded plant heights, two maximum cross diameters of the canopy projection, canopy area, and number of cones (cone crop size). We harvested 50 cones from each plant to measure their diameter, length, mass, number of seeds, average seed mass, pulp mass and number of seeds per cone. We used the manually depulped seeds (*n* = 45 560 seeds) to estimate individual seed viability for each plant by a flotation test. Finally, we estimated the nutritional characteristics of the pulp (ash, protein, fibre and lipids) using standard laboratory procedure (see electronic supplementary material, table S2 for details on measurements and procedures) for 82 of the 105 plants for which sufficient pulp was available.

### (d) Modularity analysis

We computed network modularity for the individuals–species adjacency matrix to analyse whether there was an interaction turnover along the colonization gradient. This analysis assigned all individual junipers and frugivore species to their corresponding modules based on distinctly connected subsets of nodes in the network [24]. We calculated modularity and module composition using the *computeModules* function of the *bipartite* package in R software (steps = 1000, tolerance = 1 × 10^−10^), with the Beckett algorithm [45,46]. Statistical significance of network modularity was estimated by comparing the observed network with 100 random networks generated by the *vaznull* null model (calculating the *z score* to obtain a *p-value*). This null model preserves network connectance and reshuffles interactions without keeping the marginal totals fixed. In order to test if the composition of the modules was independent of the stand identity, we quantified the number of plants in each stand provenance per module. We computed Pearson's chi-squared test of independence on a two-way contingency table (stand × module) to test whether the module membership was independent of stand identity. This approach allowed us to assess whether any of the modules in the network had an over- or under-representation of individuals of a given stand. Additionally, we extracted *c* and *z* values from the output of the *computeModules* function to explore the topological role of plants and animals. In the modularity analysis, *c* and *z* provide estimates of the among-module connectivity (fraction of interactions with partners in different modules) and within-module degree (fraction of interactions with partners in the same modules), respectively [24].

### (e) Plant traits in modules (LDA)

In order to determine the role of inter-individual variability on the modular structure, we computed a linear discriminant analysis (LDA). We set intrinsic plant traits and neighbourhood context as independent variables, and module affiliation as a categorical dependent variable. LDA allows us to determine if there is a distinct combination of plant characteristics that differentiates the plants in each module of the network. Before computing the LDA, we removed correlated variables by conducting a variance inflation factor analysis (VIF, threshold = 3) [47]. We identified variables with the highest relevance in the discriminant function based on the Wilks' lambda criterion. This analysis could not be performed on plants with missing traits (see above). Two LDA analyses were computed in parallel in order to assess whether nutritional traits could play a role in the module assignment. The main LDA was fitted with the 105 focal plants without chemical trait data. The parallel LDA analysis consists of a subset of 82 plants and two homologous analyses including and excluding pulp nutritional data. We used the two 82-plant LDAs to examine whether or not the nutritional characteristics might be important in the configuration of the modules (see ‘Parallel LDA analyses’ section in the electronic supplementary material). We computed LDA with the *lda* function in the ‘MASS’ R package [48] and Wilks' lambda with the *greedy.wilks* function in *klaR* R package [49].

### (f) Functional consequences: individual contributions to the seed rain

We estimated the number of seeds successfully dispersed by each individual juniper based on their frugivore assemblage composition and phenotypic reproductive traits. For each plant, we weighted the number of specific visits by the consecutive factors: frugivore feeding rate (cones consumed/visit), individual cone seediness (seeds/cone), frugivore seed treatment (% undamaged seeds) and individual seed viability (details in electronic supplementary material, figure S5 and electronic supplementary material, table S3). For example, if we recorded five visits of *V. vulpes* (feeding rate*_V. vulpes_* = 7.4 cones/visit; undamaged seeds*_V. vulpes_* = 97%) on plant C106 (seediness_C106_ = 5.2 seeds/cone; viability_C106_ = 21%), we estimated the number of viable seeds dispersed by *V. vulpes* in C106 focal plant as follows: 5 x 7.4 x 5.22 x 0.97 x 0.21 = 39.3 viable seeds dispersed. This approach yielded an estimate of the number of seeds dispersed by each frugivore for each focal juniper, and therefore the contribution of each individual plant to the pooled seed rain. To assess the role of the frugivore assemblage and individual traits in seed contribution, we fitted a multiple linear regression model. We built this model using the number of seeds dispersed by each frugivore species as the dependent variable (log-transformed). As predictor variables we used visits by frugivores grouped by functional groups (except for *T. philomelos*) and plant reproductive traits (seed viability and seeds/cone). Our model selection was based on AICc, which accounts for small sample sizes. The relative contribution of predictor variables was estimated by the *relaimpo* R package, which is based on *R*^2^ partitioned by averaging over orders [50]. We used simple linear models and Tukey's *post hoc* tests to analyse differences in individual seed contributions (log transformed) between modules and between stands. To detect those individuals with an outstandingly high or low contribution within each stand, we estimated the individuals' *z*-score. These *z*-scores were estimated as how many s.d. units an individual's contribution to the seed rain deviates above or below the stand mean. Finally, we used simple linear regressions to explore the relationship between individual seed contributions (log-transformed) and both plant size (a proxy of plant age) and network-node centrality (both log-transformed).

## 3. Results

### (a) *Juniperus phoenicea* frugivore assemblage

Overall, we recorded 3911 individual plant–frugivore interactions: 2628 by DNA-Barcoding analyses and 1283 by phototrapping. By standardizing these visits by sampling effort and extrapolating to the overall reproductive episode, we estimated a total of 109 988 visits to the 105 focal plants over the two seasons. Among stands, the estimated number of visits increased when approaching the colonization front (MAR = 14 555; OJI = 40 623; COL = 54 810). We recorded visits by frugivorous animals in all focal plants, with an average of 1047 ± 115 (mean ± s.e.) visits per individual. We found 12 frugivore species consuming cones of *J. phoenicea* (table 1): four thrush species *(Turdus philomelos, T. iliacus, T. merula* and *T. torquatus*), Iberian magpie (*Cyanopica cooki*), two warblers and the European robin (*Curruca melanocephala, Sylvia atricapilla* and *Erithacus rubecula*, respectively) and four mammals (*Oryctolagus cuniculus*, *Vulpes vulpes, Meles meles* and *Genetta genetta*). Yet, only three species accounted for 94% of the visits (*T. philomelos* = 76.27%, *E. rubecula* = 11.47% and *T. merula* = 6.18%).

**Table 1.** Frugivore species recorded visiting and feeding on cones of *Juniperus phoenicea*. The table shows the network module assignment, the visits detected in each of the stands (MAR = mature-stand; OJI = intermediate-maturity stand; COL = colonization-front), the total number of visits and the consumption rate per visit. The estimated number of seeds dispersed per frugivore and their relative contribution to the seed rain are also shown. Species within each module are ranked by the estimated number of dispersed seeds (decreasingly).

frugivore species	common name	functional group	module	no. visits				feeding rate (cones /visit) [± s.e.]	no. estimated dispersed seeds [ ± s.e.] (%)
				MAR	OJI	COL	total		
*Turdus philomelos*	song thrush	medium-sized bird	A	9277	29 322	45 291	83 890	4.37 ± 0.7	723 408 ± 116 725 (82%)
*Genetta genetta*	common genet	medium-sized mammal	A	0	13	52	65	2.27 ± 0.2	363 ± 39 (0.04%)
*Turdus merula*	blackbird	medium-sized bird	B	1119	3001	2677	6797	4.42 ± 0.45	56 005 ± 6014 (6.33%)
*Erithacus rubecula*	robin	small-sized bird	B	1998	5247	5368	12 613	1.25 ± 0.13	30 783 ± 3201 (3.48%)
*Vulpes vulpes*	red fox	medium-sized mammal	B	312	296	802	1410	7.4 ± 0.54	19 935 ± 2116 (2.25%)
*Oryctolagus cuniculus*	European rabbit	small-sized mammal	C	1353	2240	27	3620	9.06 ± 0.59	46 981 ± 4265 (5.31%)
*Turdus iliacus*	redwing	medium-sized bird	C	65	229	76	370	5.18 ± 0.4	3251 ± 251 (0.37%)
*Meles meles*	European badger	medium-sized mammal	C	37	9	6	52	19.83 ± 2.21	2070 ± 231 (0.23%)
*Curruca melanocephala*	Sardinian warbler	small-sized bird	C	92	213	503	808	0.62 ± 0	876 ± 0 (0.1%)
*Cyanopica cooki*	Iberian magpie	medium-sized bird	C	213	0	0	213	1.76 ± 0	428 ± 0 (0.05%)
*Turdus torquatus*	ring ouzel	medium-sized bird	C	37	0	8	45	5.42 ± 0.28	326 ± 17 (0.04%)
*Sylvia atricapilla*	blackcap	small-sized bird	C	52	53	0	105	1.33 ± 0.33	271 ± 101 (0.03%)

### (b) Module composition

**Figure 1.**
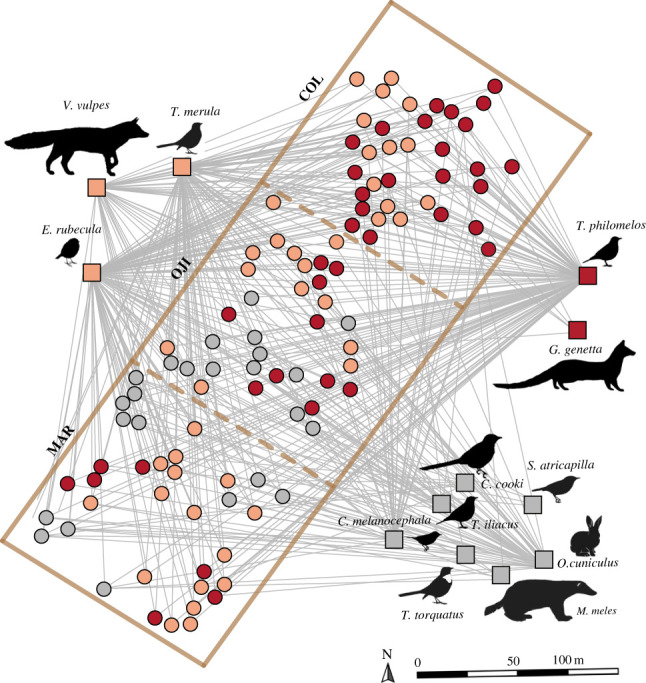
Individual-based frugivory network of *Juniperus phoenicea* along a range expansion gradient. Dots represent individual plants and squares represent frugivore species. As a spatially explicit network, the location of the plants within the panel for each stand corresponds to their actual georeferenced positions. The dotted lines represent a fictitious separation, as the stands are distant from each other (see electronic supplementary material, figure S3b). Frugivore visits to the focal plants (network links) are represented by grey lines. The colours of the network nodes represent the modules to which the individual plants and animals belong (A = red; B = salmon; C = grey). The three rectangles illustrate the three different study stands along the expansion gradient, from a mature forest area to the colonization front (MAR = mature stand; OJI = intermediate-maturity stand; COL = colonization front stand).

The individual-based frugivory network structure (an individual-species weighted bipartite network) was significantly modular (*p*-value < 1 × 10^−5^), with three densely connected groups of individual plants and animal species (figure 1, table 1). The stand membership of the plants within the modules differed from a random composition (Pearson's *X^2^* = 22.12, *d.f.* = 4, *p*-value = 0.0001), revealing a modular organization, highly concordant with the expansion gradient. That is, some modules were significantly over- or under-represented by plants from certain stands (e.g. plants in the colonization front were over-represented in module A, but module C was under-represented). There was a dominant module including most plants from the colonization front (A), a heterogeneous one with a mixed composition (B), and a third module associated with plants from the two most mature stands (C; figure 1). The first module (A) consisted of 39 focal plants linked to *T. philomelos* and *G. genetta*. *Turdus philomelos* was the main frugivore, which accounts for virtually all interactions in the module. This module was found to be significantly overrepresented by plants from the colonization front (COL = 22, OJI = 10, MAR = 7). The second module (B) was the largest module, formed by 42 individual junipers and three secondary frugivore species in the network (*V. vulpes*, *T. merula* and *E. rubecula*). Module B included a similar number of plants from the three stands (COL = 13, OJI = 13, MAR = 16). The last module (C) was the smallest one and included 24 plants and the less frequent frugivorous species (*T. iliacus, T. torquatus, C. melanocephala, S. atricapilla, O. cuniculus, C. cooki* and *M. meles*). In this module, plants from the colonization front were found to be significantly under-represented (COL = 0, OJI = 12, MAR = 12).

### (c) Plant traits in modules

We found that individual phenotype and especially neighbourhood attributes could partially predict the module membership of individual plants (electronic supplementary material, figure S6). In the LDA analyses, the two linear discriminant functions with the highest discriminating power correctly assigned each plant to module of origin in 62.85% of the cases. These discriminant functions included two neighbourhood-related factors (neighbourhood density and fecundity) and one cone trait (cone diameter; electronic supplementary material, table S4). By computing these analyses in parallel with the subset of 82 plants with available pulp nutritional data, we found that the most variable chemical trait (pulp's ash content (%); electronic supplementary material, figure S7) was also a useful trait for correctly assigning the module provenance (electronic supplementary material, table S5). Although some traits were effective in assigning plants to each module, the degree of overlap between module and phenotypes was important (electronic supplementary material, figure S6). For example, high neighbourhood density together with high values of ash concentration differentiated the module C plants from those of modules A and B. Neighbourhood crop size and cone diameter acted in the opposite direction, separating some of the plants from module A (with highly productive neighbourhoods and bigger cones) from plants of modules B and C. Finally, the plants in module C occupied a much smaller phenotypic space in the LDA plot, which indicates that the OJI and MAR plants (which compose this module) that interact with this subset of animals share similar characteristics.

### (d) Functional consequences: individual seed contribution

**Figure 2.**
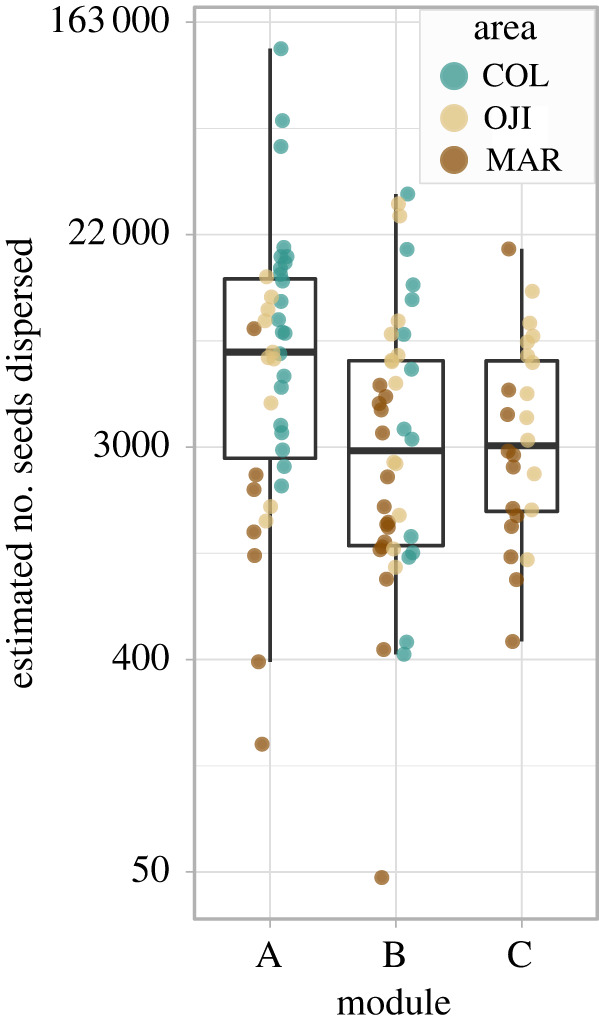
Estimated number of seeds dispersed by each individual plant in the three network modules. Each dot represents an individual plant and the colours illustrate the stand provenance. Note the log scale on the *y*-axes to improve visualization.

We estimated a total of 884 697 dispersed (viable) seeds from the 105 focal plants during the two sampling seasons. *Turdus philomelos*, which consistently showed a marked role as a connector (within and among modules) of the interaction network (electronic supplementary material, figure S8), accounted for 82% of the dispersed seeds. The remaining 18% of seeds were mainly dispersed by *T. merula* (6,3%), *O. cuniculus* (5,3%), *E. rubecula* (3,5%) and *V. vulpes* (2,3%). The number of seeds estimated to be successfully dispersed by individual junipers differed significantly between modules (linear model; *R*^2^ = 0.07, F = 4.72, *d.f.* = (2, 102), *p* = 0.01, figure 2). Plants in module A contributed more to the seed rain than plants from modules B and C (Mean ± SE, A = 13 441 ± 2098; B = 5888 ± 859; and C = 4718 ± 628 seeds). Comparing between pairs of modules, the Tukey test revealed significant differences between seed rain contributions of plants from module A and B (*p*_A-B_ = 0.01, *p*_A-C_ = 0.08, *p*_B-C_ = 0.93). We also detected significant differences in the estimated number of dispersed seeds between stands, increasing from the more mature stands to the youngest colonization front area (mean ± s.e., MAR = 2724 ± 372; OJI = 7285 ± 1066; COL = 15 268 ± 2361; Linear Model; *R*^2^ = 0.24, F = 7.22, *d.f*. = (2, 102), *p* = 3.61 × 10^−7^). Comparing between pairs of stands, a Tukey test revealed that the seed contribution by MAR plants was significantly lower than those of OJI and COL (both with *p* < 0.0001), with no significant differences between OJI and COL (*p* = 0.42).

Frugivore assemblage composition strongly determined the number of dispersed seeds (table 2). The fitted model accounted for 75% of the variation in individual seed contribution. *Turdus philomelos* visits had a positive and significant effect, explaining 29% of the variance in the model. Plant reproductive traits also had significant effects, especially individual seed viability, which accounted for more than 17% of the variance in the model. Although less important in the model, visits by small-sized birds and mammals also had a positive and significant effect on individual plant contribution to the seed rain.

**Table 2.** Multiple linear regression model summary showing the effects of frugivore functional groups visits and plant reproductive traits on individual seed contributions. In addition to the linear regression coefficients for each variable, *t*-values, *p* values and the percentage of explained variance are also shown.

	estimate ± s.e.	*t* value	*p*	variance explained
frugivore species				
intercept	4.878 ± 0.538	9.066	<0.001	
*Turdus philomelos*	0.00068 ± 0.0001	7.520	**<0**.**001**	29.0%
medium-sized birds	−0.00003 ± 0.0008	−0.039	0.968	8.5%
small-sized birds	0.0062 ± 0.00046	2.317	**0**.**022**	9.3%
mammals	0.0032 ± 0.00081	3.962	**<0**.**001**	5.4%
no. seeds per cone	0.23 ± 0.10	2.956	**<0.001**	6.2%
seed viability	0.031 ± 0.0045	6.782	**<0.001**	17.1%

**Figure 3.**
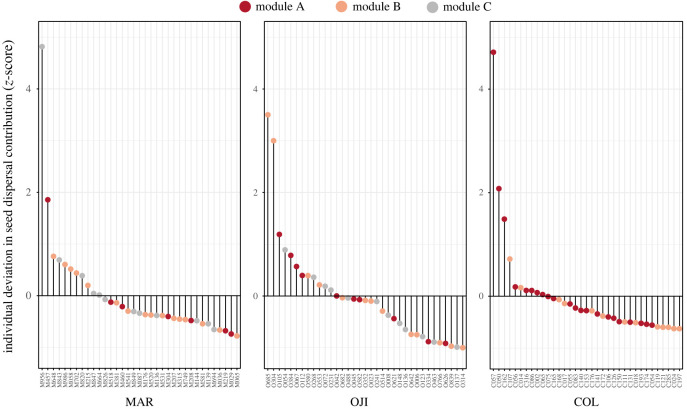
Ranked *z*-scores representing the deviation of each individual juniper from the stand mean number of seeds dispersed, i.e. how each individual contribution to the total seed rain deviates from the stand mean along the natural expansion gradient (MAR = mature stand; OJI = intermediate-maturity stand; COL = colonization front stand). Points represent individual plants and colours indicate network module assignment. Note that a more homogeneous normal distribution in the contribution would be represented with half of the individuals above the mean and half below the mean (OJI case), while a strongly skewed distribution with some outliers would result in a strongly truncated line (COL case).

**Figure 4.**
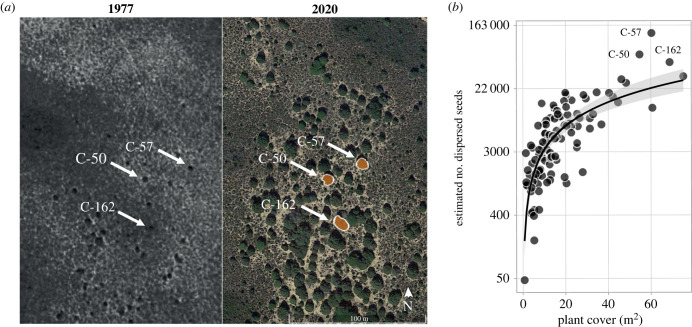
(*a*) Comparison between 1977 and 2020 aerial images of the studied colonization front stand (COL). The three individual plants marked are the individuals with an outstanding contribution to the seed rain. This contrast of images demonstrates that these now highly fecund individuals were pioneer individuals already established at this colonization front in 1977. We have validated the correct individual assignment by overlaying geographical coordinates, together with *in situ* spatial triangulation based on fixed landmarks. The historical image of 1977 comes from plane flights called ‘*Vuelo Interministerial‘* carried out by the Instituto Geográfico Nacional (available at: https://fototeca.cnig.es/fototeca/). The 2020 satellite image is available in Google Earth Pro (see https://www.google.com/intl/es/earth/versions/#earth-pro). (*b*) Relationship between the plant canopy cover and the number of estimated dispersed seeds for each plant. The dark grey line represents the fitted log–log linear regression and the grey area spans the slope confidence interval at 95%. Note the log scale on the *y*-axes to improve visualization. Individual canopy cover here is used as a proxy for plant age. Plant individuals identified in A are indicated.

We found a markedly uneven contribution of juniper individuals to the pooled estimate of dispersed seeds within stands (figure 3). Consistently among stands, few individuals deviated markedly, by several standard deviations, above the stand mean contribution of dispersed seeds (figure 3). However, in the colonization front (COL), this ranked distribution of individual contributions was markedly truncated, with very few individuals having a disproportionately high contribution to the pooled seed rain: three individuals in COL accounted for almost 50% of the seeds dispersed. By contrast, the individual contributions in the intermediate and mature stands were more even, with nine and six individual junipers contributing greater than 50%, respectively. Concordingly, these three individuals occupied a marked central role in the network topology (electronic supplementary material, figure S9), with an important function both within their module and also between modules (electronic supplementary material, figure S8). We explored the relationship between the contribution to pooled seed rain and the size of the individuals. Using the individual cover as a proxy of plant-age, we detected a positive and significant relationship between individual cover and its seed contribution (linear model; *R*^2^ = 0.60, *F* = 155.80, *d.f.* = (1, 103), *p* = 2.2 × 10^−16^; figure 4*b*), revealing a strong size effect.

## 4. Discussion

Frugivore-mediated seed dispersal plays a crucial role in the expansion of plant ranges [9], resulting in demographic consequences at both local and regional scales [51]. The varying outcomes of frugivore interactions with different species can be linked to demographic effects, which in turn can lead to population-level consequences, including range expansion or retraction. Our empirical findings demonstrate a turnover in frugivory interactions across a local gradient of natural expansion, with consequential effects on variable individual contributions to the seed rain. The entire interaction network was highly structured, featuring a distinct set of modules comprised of individual plants that matched their stand provenance and were closely associated with the natural expansion gradient. This indicates that during range expansion events, plant–frugivore interactions are not only shaped by stand characteristics, but they also influence distinct outcomes of the seed dispersal process, such as variable levels of individual plant contributions to range expansion. Our results underscore the importance and interdependence of this mutualism in promoting natural population expansion.

### (a) *Juniperus phoenicea* frugivore assemblage

The seed disperser assemblage of *J. phoenicea* was dominated by thrush species and complemented with small birds and mammals. Our results are consistent with those reported previously for other Mediterranean junipers [37–39]. However, the extensive sampling effort of this study and the combination of molecular and camera-trap techniques allowed us to describe this assemblage at the individual plant scale. For instance, we were able to detect that *Erithacus rubecula* had a significant importance (3.5% of dispersed seeds and 11.5% of total recorded visits), despite the weak trait-matching between cone size (mean [range], cone diameter = 9.3 mm [6.2–12.6]) and the *E. rubecula* gape size (7.8 mm). It may be that *E. rubecula* feeds on the smaller cones and/or on cones already handled by *Chloris chloris*, a granivorous bird species that pecks and opens the cone to feed on the seeds [8]. The European rabbit (*Oryctolagus cuniculus*), which is seldom considered as a legitimate disperser (but see [52]), was the third most important *J. phoenicea* seed disperser, surpassing larger mammals. Given the limited mobility of rabbits compared to the other larger mammals recorded, their contribution as dispersers probably occurs in the local regeneration of mature areas, in a similar way to the role of small passerines. *Vulpes vulpes* also played an important role, especially on the colonization front, which is interesting because of its potential for long-distance dispersal (LDD) in juniper species [38]. Sampling at this detailed level has allowed us to unravel how the twelve seed disperser species markedly reshape their interaction patterns along the plant range expansion gradient.

### (b) Network reconfiguration along the colonization gradient

We found a direct relationship between the composition of interaction modules and different stages of the colonization chronosequence, with the natural expansion gradient serving as a landscape template that shapes these interactions. Previous research has also shown that module composition in individual-based seed dispersal networks is influenced by habitat preferences. For instance, Friedemann *et al.* [20] found a consistent trend in the modular structure of neotropical palm-seed dispersal networks along an elevational gradient of rainforest habitats. In a similar framework, studying the Brazilian pepper, Vissoto *et al*. [16] also found a habitat-dependent module composition between frugivores and individual plants. Our findings partially support that landscape heterogeneity leaves a distinct signal in the composition of plant–frugivore interactions, which is directly mediated by the habitat preferences of frugivores and local species-specific abundances. Additionally, we observed that both neighbourhood physiognomy and its surrounding fruit availability play a role in driving variation in frugivore visitation [21,53,54]. Using LDA, we found that neighbourhood density helped in discriminating mature plants in module C from those in the other two modules. We also found that most of the frugivores in the periphery of the network, where they play a marginal role in seed dispersal, were mostly found in module C, which includes plants from the mature and intermediate stands. Species such as *O. cuniculus*, in this module, may act as habitat specialists, selecting stands with high vegetation cover and acting as module hubs. Module B species appear to structure their interactions in a habitat-independent way, as they are closely linked to plants along the entire gradient. Interestingly, module B is the only module composed exclusively by non-migratory species. Given established populations in the area, individuals of these resident species may generalize in different juniper woodland configurations. Finally, neighbourhood fecundity helped to better distinguish plants in the colonization front module, where the dominance of *T. philomelos* has the greatest consequences for the dispersal and expansion of *J. phoenicea*. Although this species interacts with all the focal plants in the study, *T. philomelos* dispersed seeds mainly from the colonization front (MAR = 8%, OJI = 27%, COL = 65%). The song thrush, which interacts with plants from the three modules and the three stands, may be identified as a module-connector (electronic supplementary material, figure S8) as reported for similar thrush species in individual-based seed dispersal networks [16,20]. The preference of song thrush for individuals from the colonization front shows that this species is able to adapt to different landscapes, especially isolated plant configurations and stands outside the juniper forest.

Physical and chemical cone traits were also relevant for differentiating between modules. For example, cone size separates plants of module A, with larger cones on average, from those of the other modules. This result makes sense because modules B and C comprised the smaller bird species, which may be actively selecting plants with smaller cones and thus configuring modules accordingly. In terms of the nutritional characteristics of the pulp, only the ash content exhibited a good discriminatory capacity for module membership, and was useful in differentiating module C plants from those of the other modules. The ash concentration is closely related to the mineral and trace element contents of the pulp [55]. Some frugivores associated with module C, which are closely linked to these mature forests, seemed to be selectively choosing plants with mineral-rich nutrient balances to supplement their diets more frequently than other more generalist frugivore species. This is likely due to the presence of rabbits as dispersers in this stand and the fact that herbivores actively seek mineral-rich foods to contribute to balancing sodium depletion and reducing toxic effects of secondary compounds [56].

Results with the mutualistic seed dispersers contrast with those reported for antagonistic networks in a previous study of the same set of *J. phoenicea* individuals, where phenotypic traits were more explanatory of interaction topologies than neighbourhood attributes [8]. Because neighbourhood attributes are closely associated with stand provenance (electronic supplementary material, figure S10), it is difficult to discern their unique effects on module structuring. These results are consistent with hierarchical levels of preference patterns for foraging frugivores [44], where animals may first select specific neighbourhood physiognomies (patches), then individual plants, and then fruits based on specific fruit traits and their variation among plants. The assemblage differences described here have consequences for the dispersal success of individuals, and unravel how they add up to determine the formation of colonization fronts.

### (c) Functional consequences: individual contributions to the seed rain

Our results suggest that substantial variation in interaction mode among plants in different modules translates into distinct individual contributions to the seed rain, and thus to the potential for *in situ* recruitment and colonization. The fact that the module dominated by the best disperser, whose visits mostly determine individual seed contribution, was over-represented by individuals from the youngest stand indicates a maximized dispersal service at the colonization front. As expected, seed viability also played a very important role in individual seed contribution. This was due to the marked variability in seed viability, which is well known for most juniper species [57,58]. The colonization front includes plants with the highest seed viability, matching those with an outstanding seed contribution (electronic supplementary material, figure S11). The physiognomy of these young stands (characterized by ample open spaces and taller plants) may favour the quality of pollination and the fecundity of these individuals, ultimately increasing their cone crop size, seed filling and attractiveness for frugivores.

### (d) Range expansion processes

The expansion of local plant population ranges is driven by individual contributions to the seed rain and the resulting consecutive demographic consequences. Fundamentally, a release of dispersal limitation constraints [51,59] may trigger a substantial advance of colonization, especially when the effect of mutualistic interactions surpasses the effects of antagonists. Our results reveal two consistent patterns along the gradient spanning from mature stands to active colonization fronts: 1) a lack of a release from pre-dispersal antagonistic interactions in the recently established areas [8] and 2) a substantial turnover of mutualistic interactions, where dominant effective frugivores (*T. philomelos*) drive active dissemination especially at the colonization front. These results suggest a possible compensatory effect between opposite-sign interactions promoting rapid natural expansion.

Our study indicates that the expansion process of juniper stands is characterized by a highly unequal distribution of seed output contributions among different individuals. Compared to more mature and intermediate succession stands, the ranked contributions of juniper individuals at the colonization front are more unevenly distributed. Our findings reveal that almost half of the seed rain contribution is performed by only three individuals, which play a central topological role in the network (electronic supplementary material, figure S9) and have an important function within and between modules (electronic supplementary material, figure S8). These inequalities in reproductive success and resulting dispersal contributions have already been reported in many tree species [60,61] and are indicative of size and fecundity hierarchies in plant populations [62]. Additionally, our analysis using aerial flight photographs (see [36]) suggests that these individuals could be the pioneer plants that acted as founders of the colonization stand. Aerial photographs and *in situ* validation show that these three high-fecundity individuals were already established in the stands by the late 1970s (figure 4*a*), during the very early stages of the expansion (electronic supplementary material, figure S12). All these results, characterised by a few fecund individuals that seem to be the founding individuals, suggest that the expansion process does not take place homogeneously, but rather through punctuated jumps. Such a process involves the occurrence of LDD events [34], whose dynamics significantly depart from purely diffusion processes. By contrast to uniform vegetation expansion with colonization fronts acting as ‘wave fronts' [30], our results point to a markedly punctuated advance mediated by LDD events, followed by local, *in situ* regeneration from these founding individuals [33–35]. This inequality in seed dispersal may have genetic consequences for expanding populations, for example, higher genetic relatedness at the colonization fronts [35,63]. By reconstructing dispersal history based on the variability of individual seed contributions throughout the expansion gradient, our results suggest that highly mobile species such as medium-sized thrushes and red foxes may be key to LDD events, facilitating the dispersal of future founder individuals.

To gain an in-depth understanding of how plant range expansions work, future research should trace the direct link between frugivores, source mother plants and dispersal locations using molecular approaches [64]. Our individual-based approach has provided detailed information about the reconfiguration of mutualistic interactions during plant expansion, their drivers and their consequences. Furthermore, our findings can be used to expand our conclusions to other zoochorous species. From a conservation and vegetation management perspective, individual-based approaches help to unveil how key demographic responses of plants to global change through range shifts may depend critically on highly limited and distinct subsets of individual plants that demand directed protection efforts.

## Data Availability

Data and code are available in Dryad repository with the DOI: https://doi.org/10.5061/dryad.h18931zq3 [65]. The data are provided in electronic supplementary material [66].
